# Cuffless Noninvasive Continuous Blood Pressure Monitoring Using Superficial Temporal Arterial Tonometry

**DOI:** 10.1109/OJEMB.2026.3667451

**Published:** 2026-02-23

**Authors:** Ge Zhu, Zifan Jiang, Gary Strangman, Yihao Zheng, Quan Zhang

**Affiliations:** Department of Biomedical EngineeringWorcester Polytechnic Institute 8718 Worcester MA 01609 USA; Department of Biomedical EngineeringGeorgia Institute of Technology 1372 Atlanta GA 30332 USA; Neural Systems GroupMassachusetts General Hospital 2348 Boston MA 02114 USA; Department of Biomedical Engineering, Department of Mechanical and Materials EngineeringWorcester Polytechnic Institute 8718 Worcester MA 01609 USA

**Keywords:** Biomechanical modeling, continuous non-invasive blood pressure monitoring (CNIBP), superficial temporal artery, tonometry

## Abstract

*Objective:* Continuous monitoring of blood pressure (BP) is a key parameter for cardiovascular assessment and hemodynamics monitoring. Current noninvasive methods are limited by frequent calibration, motion and environmental artifacts, and delayed response to rapid BP changes. In perioperative and critical-care settings, even short-duration hypotensive episodes and rapid BP lability have been associated with adverse outcomes, motivating technologies with high temporal fidelity. This study introduces a noninvasive blood pressure monitoring technique using superficial temporal artery tonometry (STAT), which employs a biomechanics-based transfer function to improve accuracy, reduce calibration requirements, and detect rapid BP changes in dynamic conditions. *Methods:* Twenty-nine recording sessions of continuous BP monitoring were collected in human subjects (*n* $=$ 10) during rest and during handgrip-induced BP fluctuations. Measurements were recorded simultaneously using the STAT method and compared to a noninvasive reference device (Finapres/Finometer volume-clamp) and Pulse Transit Time (PTT) baseline (derived from timing features) method. *Results:* Using the Finapres/Finometer as a noninvasive reference, our method achieved a mean absolute difference (MAD) of 4.8 $ \pm $ 2.2 mmHg during rest and 6.5 $ \pm $ 3.4 mmHg during handgrips, significantly outperforming PTT, especially under dynamic conditions. *Conclusion:* BP monitoring with STAT and its biomechanics-based transfer function achieved improved detection of rapid BP fluctuations, and higher accuracy than PTT under dynamic conditions. *Significance:* STAT with biomechanics-based modeling enables real-time, robust noninvasive BP monitoring, overcoming calibration, motion, and detection limitations of current methods.

## I. Introduction

Continuous blood pressure (BP) is one of the most critical monitoring parameters during anesthesia, surgery, and critical care [1]. Research indicates that high blood pressure significantly raises the risk of cardiovascular disease, a leading cause of death. Moreover, rapid BP excursions and BP lability during anesthesia are clinically important; even short durations of intraoperative hypotension have been associated with acute kidney injury and myocardial injury, and increased BP lability has been associated with worse outcomes [2], [3]. Prolonged low blood pressure is also linked to adverse effects on the cardiovascular, pulmonary, and gastrointestinal systems [4]. Therefore, it is critical to detect BP changes rapidly to ensure timely intervention or therapy and reduce undesired BP fluctuation.

Intermittent cuff-based blood pressure measurements taken every 15 to 30 minutes lack the resolution needed to detect rapid fluctuations. This can delay recognition of critical cardiovascular events. Therefore, continuous blood pressure monitoring is essential for timely detection of hypotensive or hypertensive episodes, allowing for early intervention and reduced clinical risk [5], [6], [7], [8].

The clinical gold standard for continuous BP monitoring is invasive arterial catheterization (arterial line; a-line), involves placing a catheter pressure sensor into an artery to measure BP [9], [10]. Despite its high accuracy, it causes patients suffering from physical pains and increases the risk of postoperative infection [11]. In the United States for instance, about 80,000 blood stream infections caused by an arterial catheter are observed annually [12]. Other disadvantages of a-line BP monitoring include its high cost and requirement of trained personnel for sensor placement and BP monitoring [13]. Accordingly, a-line monitoring is typically restricted to operating rooms, intensive care units, and other high-acuity hospital environments rather than routine ambulatory monitoring [14], [15]. The limitations of traditional methods have led to the search for non-invasive alternatives that provide continuous hemodynamic data without the risks and costs of arterial line systems.

Cuffless BP approaches can be organized into (i) *sensor modalities* such as PPG, ultrasound, tonometry, and volume-clamp, and (ii) *derived features* such as pulse transit time (PTT) hold significant promise for continuous cuffless BP monitoring. PPG methods are particularly popular in wearable devices due to their simplicity and low cost, as they use optical detection to monitor blood volume changes and can be paired with ECG or other proximal timing signals to compute PTT [16]. Features of the PPG waveform, such as amplitude, slope, and dicrotic notch timing, have been combined with machine learning models for cuffless BP estimation [17], [18]. However, PPG-based monitoring is susceptible to motion artifacts, skin pigmentation variations, tissue differences, and inconsistencies in sensor contact, requiring frequent calibration to accurately capture rapid BP changes [19], [20], [21].

Ultrasound sensors track arterial wall motion and lumen dynamics using thin films; however, signal drift and sensitivity to motion limit their reliability during daily activities [22]. Therefore, the relationships between PPG/BP and ultrasound/BP require further validation before they can be consistently used in clinical settings [22], [23]. Volume-clamp systems, such as the Finometer, are considered noninvasive reference devices for short-term studies, but their cuff inflation, discomfort, and bulkiness impede long-term wear and true integration as wearable cuffless devices [24]. Tonometry directly captures peripheral arterial pressure waveforms and can provide high-fidelity waveform capture and improved dynamic tracking in some settings with less need for calibration than PPG, or ultrasound, which are more susceptible to motion artifacts and drift. Additionally, tonometry causes less discomfort compared to volume-clamp systems [25], [26], [27]. We revised this wording to avoid overgeneralization and to emphasize dynamic BP tracking and waveform fidelity rather than claiming universal superiority.

This study enhances cuffless, continuous blood pressure monitoring by integrating STAT with a biomechanics-based transfer function, improving accuracy and enabling the detection of rapid BP changes under both resting and dynamic conditions....

## II. Materials and Methods

### A. Superficial Temporal Artery Tonometry

**Fig. 1.**
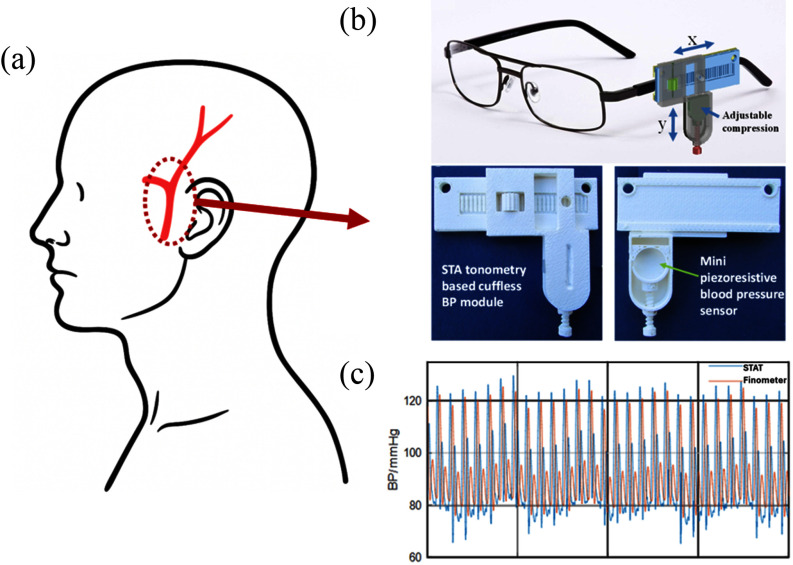
Design schematics of the STAT device (Full size figure Matin supplemental material): (A) Superficial temporal artery location, (B) STAT sensor mounted on eyeglasses, and (C) STAT data acquisition compared with a noninvasive reference continuous BP measurement device.

The superficial temporal artery (STA) offers easy localization with minimal imaging and requires an applanation pressure of under 50 g, similar to eyeglasses, ensuring comfort [28]. It has reduced motion compared to the wrist or finger and features thinner tissue layers with less facial muscle interference, making it ideal for continuous blood pressure estimation using a biomechanics-based model [29]. A lightweight, wearable STAT module enables continuous, noninvasive BP monitoring and easily mounts on eyeglasses or headphones for ambulatory use, as shown in Fig. 1. Fabricated via 3D printing, the prototype incorporates two key improvements: a mount with fine x–y axis adjustments for accurate STA localization and consistent sensor–artery alignment, and a screw-based mechanism providing tunable applanation pressure to maintain stable contact without occlusion or discomfort during extended monitoring.

The piezoresistive transducer measures pressure with a sensing diameter of 14.5 mm, a dynamic range of 0–300 mmHg, a bridge resistance of 5 k $\Omega$, and a sensitivity of 250 $\mu$V/mmHg. Initial testing indicates that most eyeglass frames provide sufficient elastic force to maintain the required applanation pressure for hours with minimal manual adjustment, supporting the feasibility of a fully wearable, passive design. The STAT sensor integrates with our patented physiological signal processing system, which synchronously records continuous BP from STAT, PTT-based cuffless measurements, Finometer BP as the reference standard, and head accelerometry for motion tracking. This configuration enables direct performance comparisons under both resting and dynamic conditions, with full methodological details available in the supplementary materials.

### B. Human Subject Data Collection

To assess individual variability and the feasibility of blood pressure (BP) measurements using the STAT device, recording sessions were conducted with 10 subjects. The experiment comprised two phases: stable BP monitoring and induced BP fluctuation monitoring. After establishing a baseline BP measurement with an ABPM, 20 minutes of resting BP data were collected as subjects minimized head motion. For the fluctuation phase, subjects performed sustained right-hand handgrip exercises at 30% of their maximal voluntary contraction in four bouts, each last 45 seconds and separated by a 30-second rest interval. This protocol was designed to elicit transient sympathetic activation and reproducible BP elevations (typically 20–40 mmHg), while incomplete hemodynamic recovery between bouts may introduce summation effects and variability in maintaining the 30% MVC target [30]. This was followed by a final resting BP recording lasting 10 minutes. Throughout the study, recordings from the STAT and Finometer were synchronized and stored for postprocessing analysis (see Supplementary material).

### C. Evaluation Metrics and Reference Definition

Performance was evaluated using mean absolute difference (MAD) consistent with IEEE 1708 guidance for cuffless devices [31]. The Finapres/Finometer volume-clamp system was used as a *noninvasive reference device* for beat-to-beat waveform comparison (not a clinical gold standard; invasive arterial catheterization remains the clinical gold standard).

MAD was calculated as:

\begin{equation*}
\mathrm{MAD} = \frac{\sum _{i=1}^{n} |p_{i} - y_{i}|}{n} \tag{1}
\end{equation*}

where $p_{i}$ is the proposed device measurement and $y_{i}$ is the reference device measurement (Finometer in this study).

For interpretability, MAD accuracy levels were compared against ANSI/AAMI SP10 [32] and the British Hypertension Society (BHS) grading [33] (Table I).

**TABLE I:** MAD Accuracy Levels Compared to ANSI/AAMI SP10 and BHS Evaluation Systems

**MAD (mmHg)**	**IEEE 1708**	**ANSI/AAMI SP10**	**BHS**
$\leq 5$	A	Pass	A
$5 < \mathrm{MAD} \leq 10$	B	Pass	B
$10 < \mathrm{MAD} \leq 15$	C	Fail	C
$> 15$	D	Fail	D

### D. Biomechanics-Based Transfer Function

**Fig. 2.**
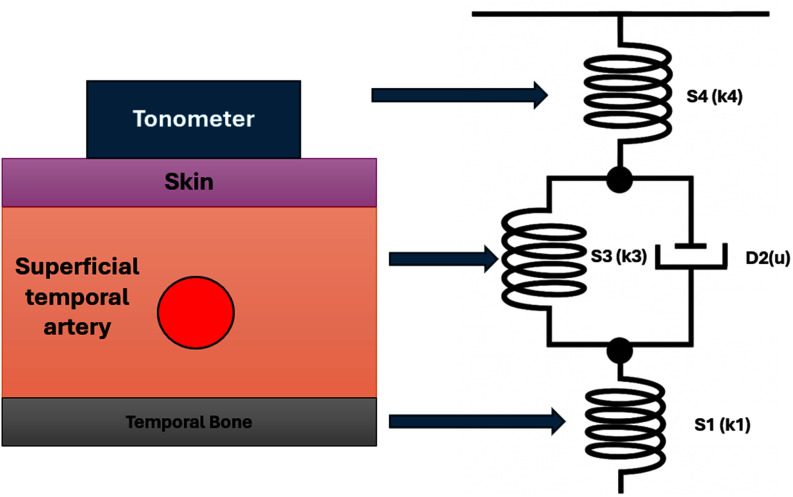
Schematic representation of the superficial temporal artery, surrounding tissues, and their mapping to the Kelvin–Voigt viscoelastic model.

The biomechanics-based transfer function for STAT data was adapted from a validated viscoelastic blood pressure model originally developed for radial artery tonometry [34], [35]. To account for the superficial temporal artery's simpler anatomy with fewer connective tissue layers, we reduced the model complexity and tested its applicability through *in silico* simulations. Using Kelvin–Voigt (KV) viscoelastic parameters from the literature (Fig. 2), we examined the relationship between internal arterial pressure ($P_{a}$) and sensor surface pressure ($P_\sigma$). Simulations demonstrated a nearly linear $P_{a}$–$P_\sigma$ relationship across physiological ranges at the superficial temporal artery, supporting the reduced model's validity.

Tonometry measures intravascular pressure by applying external pressure until the artery is optimally flattened. This eliminates circumferential stress, making the surface pressure proportional to the internal pressure. The relationship is defined by:

\begin{equation*}
P_{a} = \frac{E \cdot h}{r \cdot (1-\nu ^{2}) } \cdot \epsilon + P_\sigma \tag{2}
\end{equation*}

where $E$ is vessel wall Young's modulus, $h$ is wall thickness, $r$ is radius, $\nu$ is Poisson's ratio, and $\epsilon$ is strain.

Complementing this elastic formulation, the STA-specific biomechanics-based transfer function was expressed as:

\begin{equation*}
P_{a} (t)=\frac{G \cdot A_{\mathrm{sensor}}}{A_{\mathrm{STA}}} P_\sigma (t)+\beta \frac{dP_\sigma }{dt}+P_{\mathrm{offset}} \tag{3}
\end{equation*}

where $G$ denotes the geometric coupling efficiency, $A$ the effective contact area, $\beta$ the viscous coefficient, and $P_{\mathrm{offset}}$ the calibration offset (see supplemental material Table 1).

The artery–sensor interface was further modeled using a Kelvin–Voigt viscoelastic representation, which combines an elastic spring and viscous dashpot in parallel. The constitutive equation is:

\begin{equation*}
\sigma =k_{2} l_{2}+\eta \dot{l}_{2} \tag{4}
\end{equation*}

where $k_{2}$ represents tissue elasticity, $\eta$ the viscous damping, and $\ell _{2}$ the viscoelastic deformation.

Coupled with the series arterial spring $k_{1}$, the governing differential equation becomes:

\begin{equation*}
\left(1+\frac{k_{2}}{k_{1}}\right)\sigma +\frac{\eta }{k_{1}} \dot{\sigma }=k_{2} l+\eta \dot{l} \tag{5}
\end{equation*}

which yields the Laplace-domain transfer function and the corresponding time-domain impulse response:

\begin{align*}
H(s)& =\frac{l(s)}{\sigma (s)}=\frac{k_{1} (k_{2}+\eta s)}{(k_{1}+k_{2}) +\eta s} \tag{6}
\\
H(t)& =\frac{1}{k_{1}} \delta (t)+\frac{1}{\eta } e^{-\frac{k_{2}}{\eta } t} u(t) \tag{7}
\end{align*}

Convolution of this impulse response with the arterial pressure waveform provides the predicted sensor output:

\begin{equation*}
P_\sigma (t)=\int _{-\infty }^{t} H(t-\tau)P_{a} (\tau)d\tau \tag{8}
\end{equation*}

**Fig. 3.**
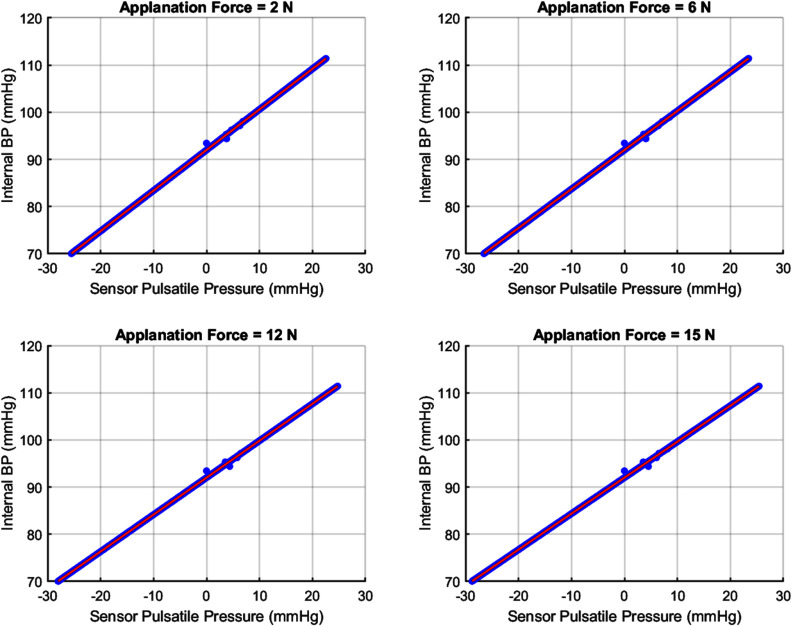
Fig. 3. Linearity between radial tonometry sensor pressure $P_\sigma$ and internal arterial pressure $P_{a}$ across physiologically relevant applanation forces. (a) Coefficient of determination ($R^{2}$) for linear regression of the pulsatile components of $P_\sigma$ and $P_{a}$ remains near unity over applied forces from 0–15 N, indicating strong linear coupling. (b) Estimated linear gain shows minimal dependence on applanation force, reflecting stable mechanical transfer. (c) Representative scatter plots at selected force levels demonstrate an approximately linear $P_\sigma$–$P_{a}$ relationship with consistent slope and negligible offset. Collectively, these results confirm that within the clinically relevant force range, the Kelvin–Voigt–based tonometry model preserves proportionality between sensor output and internal arterial pressure, supporting linear calibration assumptions.

Simulation studies with physiologically realistic viscoelastic parameters show that the Kelvin–Voigt (KV) tonometry model maintains stable arterial– sensor coupling and an approximately linear relationship between internal arterial pressure $P_{a}$ and measured sensor pressure $P_\sigma$ across clinically relevant applanation forces (0–15 N; Fig. 3). Linear regression yielded consistently high goodness of fit ($R^{2} > 0.98$), with minimal variation in slope and small offsets across force levels, indicating amplitude-independent proportionality between the two signals. This near-linear behavior supports treating the KV transfer as a first-stage, physics-informed pre-calibration that maps the raw tonometry waveform to an arterial-pressure–equivalent signal.

## III. Results

Of 29 collected recording sessions, 9 were excluded (8 due to a malfunctioning pressure sensor and 1 for excessive motion), leaving 20 valid recordings for analysis. Performance was evaluated relative to the Finometer noninvasive reference device using MAD as defined in Methods (IEEE 1708) [31].

We evaluated systolic (SBP), diastolic (DBP), and mean blood pressure (MBP) during resting and induced monitoring, using MAD as the primary metric. Finometer SBP and DBP signals showed 2-second peak noise from 60-second self-calibration, while MBP was less affected as opposing SBP and DBP peaks canceled out. Thus, MAD of MBP was selected as the main performance metric. SBP and DBP time-series and agreement analyses are provided in the Supplementary Materials.

We evaluated systolic (SBP), diastolic (DBP), and mean blood pressure (MBP) during resting and induced monitoring, using mean absolute difference (MAD) as the primary metric. Finometer SBP and DBP signals showed 2-second peak noise from 60-second self-calibration, while MBP was less affected as opposing SBP and DBP peaks canceled out. Thus, MAD of MBP was selected as the main performance metric.

### A. Accuracy of BP Measurement in Resting and Induced BP Fluctuation

Accuracy was assessed using the mean absolute difference (MAD) of SBP, DBP, and MBP between STAT, PTT, and Finometer across both resting and induced BP fluctuation phases. For resting measurements, the first 20 minutes of each valid recording (n = 20) were analyzed; STAT and PTT showed similar MAD values (Table II), with the recordings divided into 10 segments (2 min each) for analysis and visualization of agreement. The correlation and Bland–Altman plots (Figs. 5–6) confirmed comparable precision and good agreement with the reference.

**TABLE II:** MAD Values (mmHg) for SBP, DBP, and MBP Using STAT and PTT Methods. Skewness in Parentheses

**Condition**	**Method**	**SBP**	**DBP**	**MBP**
Resting	STAT	6.8$\pm$ 2.3 (0.6)	5.0$\pm$ 2.7 (2.0)	4.8$\pm$ 2.2 (1.6)
	PTT	8.6$\pm$ 3.8 (1.1)	4.3$\pm$ 1.9 (1.7)	5.3$\pm$ 2.6 (1.6)
Induced	STAT	9.6$\pm$ 4.0 (0.3)	6.9$\pm$ 4.0 (1.0)	6.5$\pm$ 3.4 (1.8)
	PTT	18.3$\pm$ 6.4 (0.3)	8.9$\pm$ 2.6 (-0.6)	11.7$\pm$ 3.6 (-0.4)

**Fig. 4.**
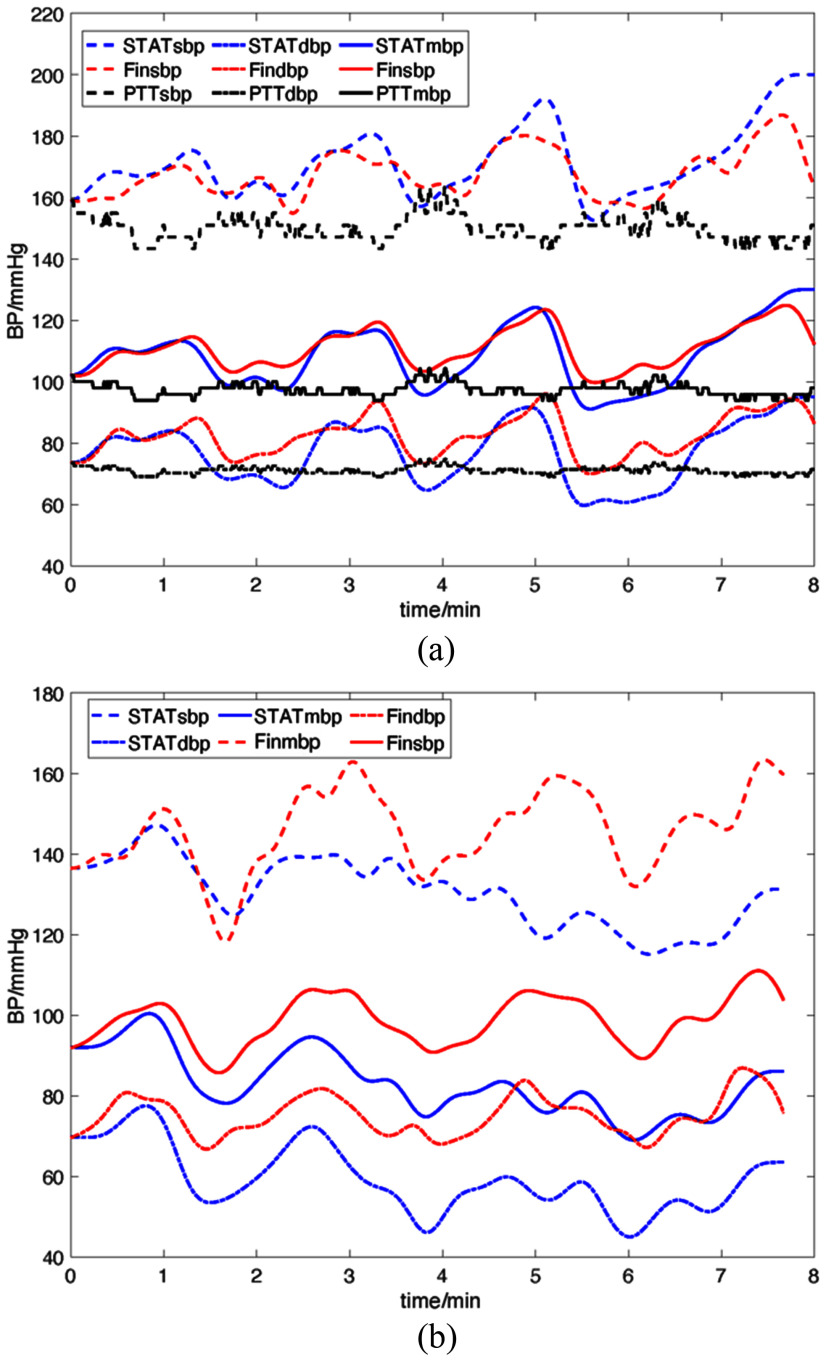
Comparison of continuous blood pressure (BP) waveforms from the superficial temporal artery tonometry (STAT) method and reference measurements. (a) STAT-derived SBP, DBP, and MBP (STAT_sbp_, STAT_dbp_, STAT_mbp_) versus Finometer (Fin_sbp_, Fin_dbp_, Fin_mbp_) and pulse transit time (PTT) estimates (PTT_sbp_, PTT_dbp_, PTT_mbp_) under resting and induced BP fluctuations. (b) STAT closely follows Finometer waveforms across all BP metrics, capturing both rapid and gradual changes with high temporal fidelity.

**Fig. 5.**
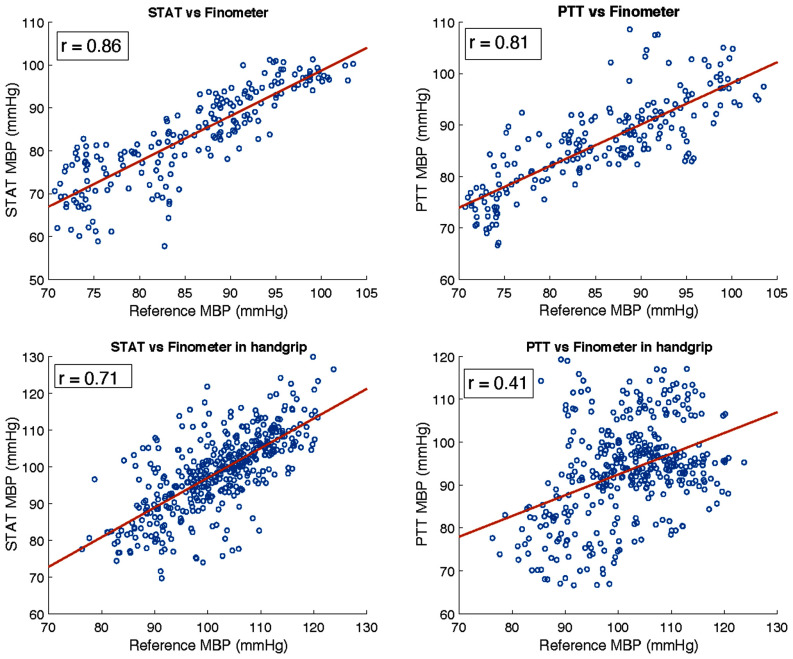
Correlation analysis of mean blood pressure (MBP) measurements between superficial temporal artery tonometry (STAT), pulse transit time (PTT), and the Finometer reference method under resting and induced conditions. Top row: Static conditions showing strong correlations between STAT vs. Finometer ($r=0.86$) and PTT vs. Finometer ($r=0.81$). Bottom row: Induced dynamic conditions with handgrip maneuvers, where STAT vs. Finometer maintained a moderate correlation ($r=0.71$), while PTT vs. Finometer showed a markedly reduced correlation ($r=0.41$). These results demonstrate the robustness of STAT for tracking rapid BP fluctuations compared to PTT-based methods.

**Fig. 6.**
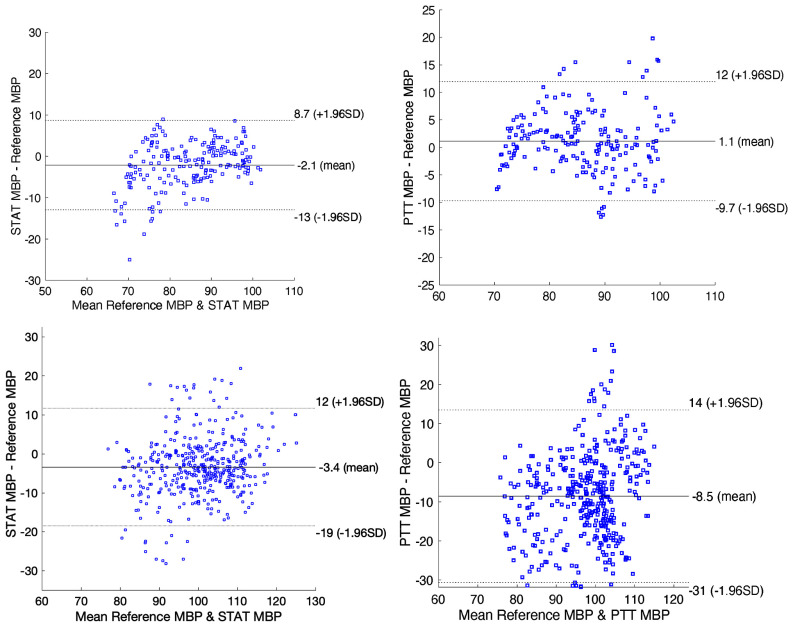
Bland–Altman analysis of MBP from STAT and PTT versus Finometer under resting (top) and handgrip-induced (bottom) conditions. Resting: STAT bias $-2.1$ mmHg (limits of agreement (LoA) $-13$ to 8.7), PTT bias 1.1 mmHg (LoA $-9.7$ to 12). Handgrip: STAT bias $-3.4$ mmHg (LoA $-19$ to 12), PTT bias $-8.5$ mmHg (LoA $-31$ to 14). STAT shows narrower limits and greater stability than PTT, especially under dynamic conditions.

For induced BP fluctuations ($\geq$20 mmHg in 14/20 recordings), recordings were split into 30 segments (15 s each). Fig. 4(a) shows that during handgrip-induced fluctuations, STAT accurately tracked amplitude and timing relative to Finometer, while PTT failed to capture these dynamics. Statistical analysis (Mann–Whitney U, $p< 0.01$) confirmed that STAT achieved significantly better performance than PTT under fluctuating conditions.

## IV. Discussion

We evaluated the STAT-based BP monitoring system against PTT-derived estimates and the Finometer noninvasive reference device under both resting and induced dynamic conditions. To our knowledge, this is the first demonstration of noninvasive continuous BP monitoring using superficial temporal artery tonometry integrated with a biomechanics-based transfer function.

Under resting conditions, STAT and PTT showed comparable agreement with the reference, indicating that timing-based surrogates can perform adequately when BP changes are gradual and vascular tone is stable. In contrast, during handgrip-induced BP fluctuations ($\geq$20 mmHg), STAT significantly outperformed PTT, consistent with the advantage of direct mechanical coupling to the arterial waveform for preserving rapid amplitude and timing changes. This improved dynamic tracking is reflected by lower MAD and tighter Bland–Altman limits.

Several limitations warrant consideration. The transfer function assumes stable sensor– tissue coupling and a simplified linear viscoelastic response; deviations in tissue properties, preload, or applanation geometry may introduce gain or offset drift not fully captured by the current model. The eyeglass-mounted form factor is sensitive to head motion, facial movement, and contact-force variability, which may limit robustness during free-living use. In addition, the study was conducted in a small cohort under controlled laboratory conditions, and the handgrip protocol may have introduced incomplete hemodynamic recovery between bouts. Finally, the current prototype requires an initial external BP calibration, a limitation shared by many cuffless approaches.

Future work will focus on improving long-term stability and robustness through contact-force sensing, mechanical stabilization strategies, and expanded validation in larger and more diverse populations.

## V. Conclusion

This study establishes STAT combined with a biomechanics-based transfer function as a feasible strategy for continuous, cuffless blood pressure monitoring. Compared with a pulse transit time baseline and a noninvasive reference device, STAT demonstrated improved capability to capture rapid BP fluctuations while maintaining comparable performance under resting conditions.

These findings underscore the value of mechanically coupled sensing for applications requiring high temporal fidelity, where delayed or smoothed surrogate measures may be insufficient. Although the current implementation employs a head-mounted form factor, its ability to detect acute hemodynamic changes positions STAT as a promising approach for monitored clinical environments, such as perioperative or critical-care settings, where continuous and responsive BP assessment is essential.

## Supplementary Materials

Supplementary materials includes experimental setup, data collection, device design, data post processing, and workflow diagram of the data processing algorithm.

## Acknowledgment of AI Use in Editing

The authors acknowledge the use of an AI-based language editing tool (Grammarly) for grammar correction and improvement of language clarity in this manuscript (main text and supplementary materials). The AI tool was used solely for editorial assistance. All study design, research content, experimental protocols, data collection, analysis, interpretation, and conclusions are the original work of the authors.

## Author Contribution

GZ, ZFJ: Designed the experiments, performed data collection, and data analysis. GZ also responsible for data interpretation, figure preparation, and manuscript preparation.

YHZ: Contributed to the conception of the research, data interpretation, and manuscript review.

QZ: Conceived the research, designed experiments, performed data collection, and prepared and revised the manuscript.

All authors contributed to the article and approved the submitted version.
